# Multicenter safety study of mFOLFOX6 for unresectable advanced/recurrent colorectal cancer in elderly patients

**DOI:** 10.1186/1756-9966-28-109

**Published:** 2009-08-07

**Authors:** Shinichi Sugimoto, Kuniyuki Katano, Akiyoshi Kanazawa, Hiroshi Yoshimura, Akihiko Kidani, Hiroshi Takeda, Masato Makino, Nobuhiro Ozaki, Tsuneo Tanaka, Masahide Ikeguchi

**Affiliations:** 1 Department of Surgery, Shimane Prefectural Central Hospital, Izumo, Japan; 2 Division of Surgical Oncology, Faculty of Medicine, Tottori University, Yonago, Japan; 3 Department of Gastroenterological Surgery, Osaka Red Cross Hospital, Osaka, Japan; 4 Department of Digestive and General Surgery, Faculty of Medicine, Shimane University, Izumo, Japan; 5 Department of Surgery, Nakagawa Hospital, Fukuoka, Japan; 6 Department of Surgery, Nojima Hospital, Kurayoshi, Japan

## Abstract

**Background:**

Combination chemotherapy with oxaliplatin plus 5-fluorouracil/leucovorin (FOLFOX) has become a standard regimen for colorectal cancer. An increase of adverse events with combination chemotherapy is predicted in elderly patients, and it remains controversial whether they should receive the same chemotherapy as younger patients. Accordingly, this study of modified FOLFOX6 (mFOLFOX6) therapy was performed to compare its safety between elderly and non-elderly patients.

**Methods:**

We prospectively studies 14 non-elderly patients aged <70 years old and 8 elderly patients aged ≥ 70 years with unresectable advanced/recurrent colorectal cancer who received mFOLFOX6 therapy during the period from March 2006 to March 2007. Adverse events and the response to treatment were compared between the elderly and non-elderly groups.

**Results:**

The main adverse events were neutropenia and peripheral neuropathy, which occurred in 62.5% (≥ grade 3) and 87.5% (≥ grade 1) of elderly patients. The grade and frequency of adverse events were similar in the elderly and non-elderly groups. In some patients with neutropenia, treatment could be continued without reducing the dose of oxaliplatin by deleting bolus 5-fluorouracil. A correlation was found between the cumulative dose of oxaliplatin and the severity of neuropathy, and there were 2 elderly and 3 younger patients in whom discontinuation of treatment was necessary due to peripheral neuropathy. The median number of treatment cycles was 10.0 and 9.5 in the non-elderly and elderly groups, respectively. The response rate was 60.0% in the non-elderly and 50.0% in the elderly group, while the disease control rate was 100% and 83.3% respectively, showing no age-related difference.

**Conclusion:**

mFOLFOX6 therapy was well-tolerated and effective in both non-elderly and elderly patients. However, discontinuation of treatment was sometimes necessary due to peripheral neuropathy, which is dose-limiting toxicity of this therapy.

## Background

A high response rate has been reported for FOLFOX therapy that includes oxaliplatin in patients with unresectable advanced/recurrent colorectal cancer, and this therapy is now established as one of the standard treatment option [1,2]. Since the introduction of oxaliplatin to Japan in April 2005, FOLFOX therapy has also become widely used in this country and is recommended as one of the standard treatments [3]. There are a number of versions of FOLFOX therapy among which modified FOLFOX6 (mFOLFOX6) allows more convenient administration and has been adopted by many medical institutions in association with popularization of outpatient chemotherapy. However, there have been few adequate investigations into the safety and efficacy of mFOLFOX6 therapy. A rapid increase in the incidence of colorectal cancer among elderly Japanese persons is anticipated in the future, considering the current long average life span and the increase in the incidence and mortality of colorectal cancer in Japan. However, it remains controversial as to whether the same multi-drug chemotherapy employed for younger patients should also be given to elderly patients, because an increase in the severity of adverse events is likely in the elderly due to the decline of organ function associated with ageing. Accordingly, the present study was performed to examine the safety and efficacy of mFOLFOX6 therapy in patients over 70 years old.

## Subjects and methods

### Subjects

A multicenter study on the treatment of unresectable advanced/recurrent colorectal cancer was started in 2006 by the Sanin Study Group on colorectal cancer (SSCC). To determine whether mFOLOFX6 could be used safely to treat unresectable advanced/recurrent colorectal cancer in elderly patients, the present study (SSCC-0601) was also performed by the SSCC.

Patients who met the following eligibility criteria and received mFOFOX6 therapy at any of the three participating institutions (Division of Surgical Oncology, Faculty of Medicine, Tottori University; Department of Digestive and General Surgery, Faculty of Medicine, Shimane University; and Department of Surgery, Shimane Prefectural Central Hospital) during the period from March 2006 to March 2007 were enrolled.

The protocol was approved by the institutional ethics committees and this study was carried out according to the principles of the Declaration of Helsinki and Good Clinical Practice guidelines.

The eligibility criteria were histologically proven unresectable colorectal adenocarcinoma; adequate bone marrow, liver, and renal function; Eastern Cooperative Oncology Group (ECOG) performance status (PS) <2; age >20 years at the time of enrolment; and expected survival time >12 weeks. Any previous chemotherapy (only 1 regimen was allowed) must have been completed at least 28 days before enrolment. Postoperative adjuvant therapy was not counted as prior chemotherapy. Patients with multiple malignancies, comorbidities that could influence the outcome, prior radiotherapy, pregnancy or lactation, symptomatic peripheral neuropathy, or a history of serious drug hypersensitivity were excluded. Written informed consent was obtained from all of the subjects.

### Treatment schedule

**Figure 1:**
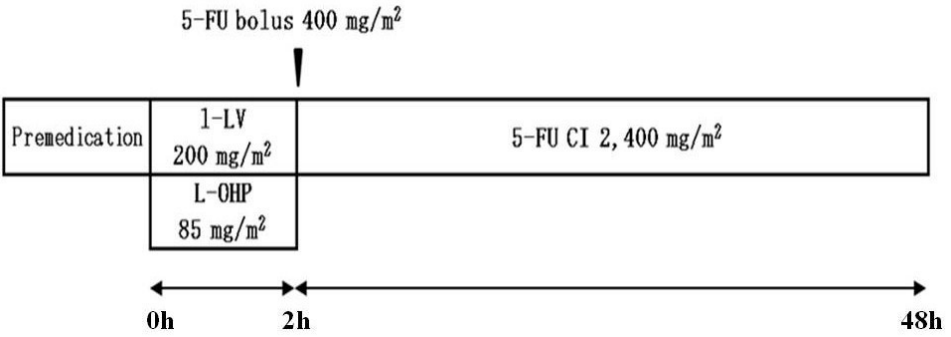
**Schedule for mFOLFOX Therapy**.

An implantable port and a disposable pump were employed so that chemotherapy could be administered on an outpatient basis. An outline of the administration method for mFOLFOX6 therapy, in which the dose of oxaliplatin was reduced from 100 mg/m^2 ^to 85 mg/m^2^, is shown in Figure 1. A 5-HT_3 _antagonist and a steroid were administered as premedication. A 2-hour intravenous infusion of oxaliplatin plus l-leucovorin was followed by bolus intravenous injection of 5-FU, after which 5-FU was administered by continuous infusion for 46 hours. An oral steroid was administered for 3 days from day 2 after the start of therapy. The duration of one cycle was 2 weeks.

With each treatment cycle, administration was only started after confirming that all of the following criteria had been fulfilled.

(1) Hematological toxicity: leukocyte count >3,000/mm^3 ^and platelet count >75,000/mm^3^.

(2) Non-hematological toxicity: Grade 2 or less according to the National Cancer Institute Common Toxicity Criteria (NCI-CTC), and Grade 1 or less for peripheral neuropathy.

(3) Even if these conditions for treatment were met, administration could be postponed at the investigator's discretion (e.g., for a rapid decrease of the leukocyte count/platelet count, occurrence of jaundice, etc).

If any of the criteria were not met, treatment was postponed. The subsequent course could be postponed for up to 21 days (excluding the scheduled day of starting administration). If administration could not be commenced during this period, the study was discontinued.

### Discontinuation of therapy

Administration was continued until any of the following criteria for discontinuation were fulfilled.

(1) The patient was judged to have progressive disease (PD), including clinical PD.

(2) Adverse events occurred that made further administration difficult.

(3) The patient did not fulfill the administration criteria and the next course of treatment could not be started by 21 days after the scheduled day of administration.

(4) A second dose reduction was considered to be necessary (Table 1).

**Table 1:** Dose-Reduction Criteria and Dose to be selected at Dose-Reduction

**Item**		**Oxaliplatin**	**5-FU (bolus)**	**5-FU (infusion)**
**Neutrophil count**	**< 500/mm^3^**	85 → 85	**400 → 0**	2,400 → 2,400
**Platelet count**	**< 50,000/mm^3^**	85 → 85	**400 → 0**	2,400 → 2,400
**Non-hematological toxicity**	≥ **Grade 3**	**85 → 65**	**400 → 300**	**2,400 → 2,000**
**Skin symptoms**	≥ **Grade 3**	85 → 85	**400 → 300**	**2,400 → 2,000**
**Peripheral neuropathy**	**Grade 2**	**85 → 65**	400 → 400	2,400 → 2,400
Acute*^1 ^laryngopharyngeal dysesthesia **(feeling of difficulty in breathing)**		**85 → 85** **Infusion time is prolonged to** 6 hours*^2^	400 → 400	2,400 → 2,400
**Peripheral neuropathy**	≥ **Grade 3**	**Discontinuation**		
**PS**	≥ **3**	**Discontinuation**		

Abbreviation: PS, performance status

*1 During the period from administration of oxaliplatin to 2 hours after completion of administration.

*2 Administration of 5-FU should not be started until the completion of administration of oxaliplatin.

(5) Peripheral neuropathy of grade 3 or 4 occurred.

(6) The PS became 3 or higher.

(7) The patient refused further treatment.

(8) The investigator judged that continuation of the study was difficult for any other reason.

### Endpoints

The incidence and severity of adverse events were assessed as the primary endpoints, while the duration of treatment, antitumor effect (response rate, tumor stabilization rate, and duration of response), and the safety and efficacy in elderly patients were assessed as the secondary endpoints. Adverse events and therapeutic efficacy were assessed according to the NCI-CTC (version 3) (Cancer Therapy Evaluation Program, NCI, Bethsada, Md., USA) and the RECIST guidelines (version 3) [4]. Extramural review was performed for judgment of the eligibility and handling of registered patients, as well as for safety and efficacy assessment.

### Statistical analysis

The chi-square test for independence, Fisher's exact probability test, and the Mann-Whitney U test were used to compare patient characteristics, treatment status, adverse events, and antitumor effect. A probability (P) value of less than 0.05 was considered statistically significant for comparisons between the younger and elderly groups. The Kaplan-Meier method was used to estimate the time to treatment failure (TTF).

## Results

### Patient profile

All of the 22 patients enrolled in this study were eligible. Their median age was 66 years (range: 39–79 years), including 14 non-elderly patients with a median age of 63.5 years (range: 39–69 years: younger group) and 8 elderly patients with a median age of 74.5 years (range: 71–79 years: elderly group). Although the elderly group had a higher incidence of colon cancer (P = 0.011), there were no marked differences of the other background factors (Table 2).

**Table 2:** Patients Characteristics

	**< 70 Years (n = 14)**	≥ **70 Years (n = 8)**	**P values**
**Age (median)**	63.5 [39–69]	74.5 [71–79]	-
**Sex (male/female)**	11/3	5/3	*0.3695
**PS (ECOG) 0/1/2**	9/5/0	7/1/0	**0.2505
**Primary tumor Colon/rectum/colorectal**	4/8/2	7/1/0	*0.011/0.052/0.3939
**Target lesions** **liver/lung/LN/peritoneum/others**	4/2/6/0/2	4/1/1/1/1	*0.291/0.709/0.161/ 0.364/0.709
**Previous surgery (+/-)**	12/2	8/0	*0.3939
**Adjuvant chemotherapy(+/-)**	4/10	2/6	*0.6305
**Previous treatment (+/-)**	1/13	1/7	*0.6060

Abbreviation: PS, performance status; ECOG, Eastern Cooperative Oncology Group; LN, lymph node.

*P values for SEX, primary tumor, target lesions, previous surgery (+/-), adjuvant chemotherapy (+/-) and previous treatment (+/-) were calculated with the use of Fisher's exact probability test. **P values for PS were calculated with the use of Mann-Whitney U test.

### Treatment status

The total number of cycles administered was 198, with a median of 10.0 cycles per patient in the younger group and 9.5 cycles in the elderly group, showing no difference (P = 0.8912 by the Mann-Whitney U test). Postponement of treatment due to toxicity occurred during 14.4% (18/125) of the treatment cycles in the younger group and 6.8% (5/73) of the cycles in the elderly group (P = 0.1907 by the chi-square test for independence).

### Adverse events

**Figure 2:**
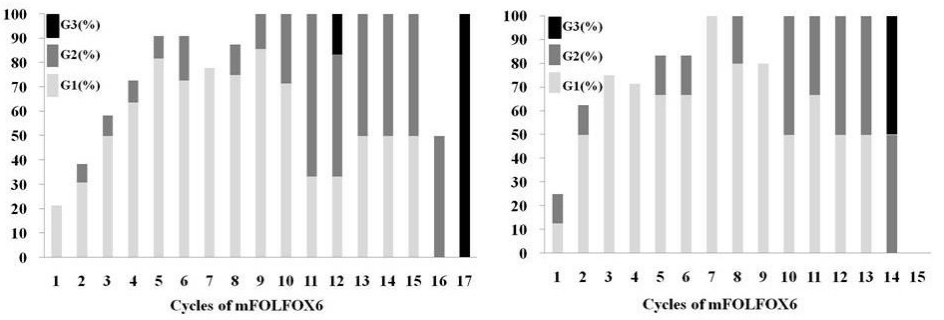
**Occurrence of Peripheral Neuropathy in younger patients (left) and elderly patients (right)**. Abbreviation: G, Grade.

Adverse events that showed a high incidence included neutropenia and peripheral neuropathy. The grade and frequency of the other adverse events were similar between the younger and elderly groups (Table 3). In 3 patients (one younger patient and 2 elderly patients) who developed grade 4 neutropenia, treatment could be continued without reducing the dose of oxaliplatin by deleting bolus 5-fluorouracil (Table 1). Peripheral neuropathy of grade 1 or more occurred at an incidence of 86.4% in the younger group and 87.5% in the elderly group (P = 0.7090), while grade 3 neuropathy occurred in 3 patients (14.3%) from the younger group and 1 patient (12.5%) from the elderly group (P = 0.7090) (Table 3). The incidence of neuropathy in relation to the number of treatment cycles is shown in Table 4. There was an increase in the incidence along with the dose of oxaliplatin, and grade 2 or worse neuropathy showed an incidence higher than 50% during the 11th cycle in the younger group and the 10th cycle in the elderly group (Figure 2).

**Table 3:** Major Adverse Events

**Grade **≥ **3**	**< 70 Years (n = 14)**	≥ **70 Years (n = 8)**	P values*
**Leukocytopenia**	2 [14.3%]	1 [12.5%]	0.7090
**Neutropenia**	4 [28.6%]	5 [62.5%]	0.1347
**Anemia**	0 [0.0%]	0 [0.0%]	-
**Thrombocytopenia**	0 [0.0%]	0 [0.0%]	-
**Nausea**	2 [14.3%]	0 [0.0%]	0.3939
**Anorexia**	1 [7.1%]	1 [12.5%]	0.6060
**Fatigue**	1 [7.1%]	1 [12.5%]	0.6060
**Stomatitis**	1 [7.1%]	0 [0.0%]	0.6363
**Hand-foot syndrome**	1 [7.1%]	0 [0.0%]	0.6363
**Peripheral Neuropathy**			
**Grade **≥ **1**	12 [86.4%]	7 [87.5%]	0.7090
**Grade **≥ **2**	6 [45.5%]	4 [50.0%]	0.5464
**Grade **≥ **3**	2 [14.3%]	1 [12.5%]	0.7090

Grades of adverse events were defined according to NCI-CTC v3.0

*P values were calculated with the use of Fisher's exact probability test.

**Table 4:** Incidence of Peripheral Neuropathy during Treatment Cycles

**< 70 Years (n = 14)**																	
**Cycles**	**1**	**2**	**3**	**4**	**5**	**6**	**7**	**8**	**9**	**10**	**11**	**12**	**13**	**14**	**15**	**16**	**17**

**Grade1**	3	4	6	7	9	8	7	6	6	5	2	2	2	1	1	0	0
**Grade2**	0	1	1	1	1	2	0	1	1	2	4	3	2	1	1	1	0
**Grade3**	0	0	0	0	0	0	0	0	0	0	0	1	0	0	0	0	1
**n**	14	13	12	11	11	11	9	8	7	7	6	6	4	2	2	2	1

≥ **70 Years (n = 8)**																	

**Grade1**	1	4	6	5	4	4	6	4	4	2	2	1	1	0	0	0	0
**Grade2**	1	1	0	0	1	1	0	1	0	2	1	1	1	1	0	0	0
**Grade3**	0	0	0	0	0	0	0	0	0	0	0	0	0	1	0	0	0
**n**	8	8	8	7	6	6	6	5	5	4	3	2	2	2	1	0	0

Grades of adverse events were defined according to NCI-CTC v3.0

*P values were calculated with the use of Fisher's exact probability test.

### Duration of Treatment

**Figure 3:**
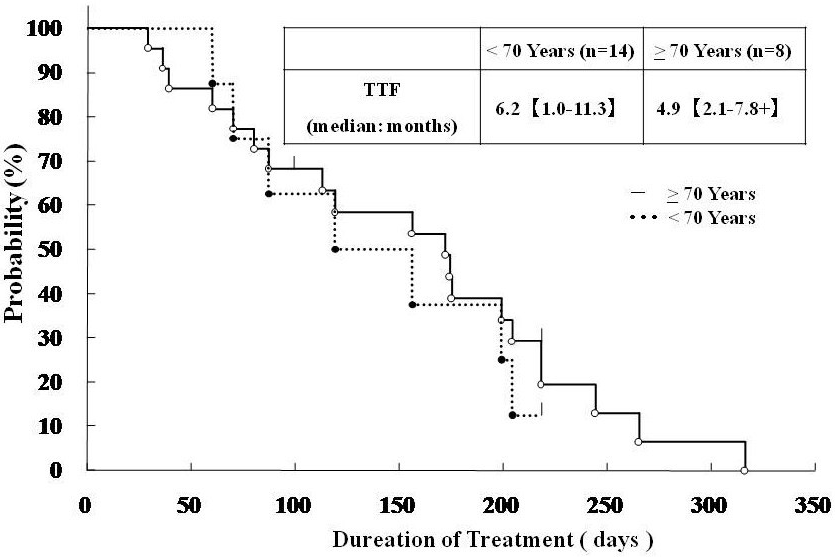
**Time to Treatment Failure (TTF)**. The Kaplan-Meier method was used to estimate TTF curves. Median value for each group is shown.

The time to treatment failure (TTF) was 6.2 months in the younger group, and 4.9 months in the elderly group, being slightly shorter in the latter group (Figure 3). The major reasons for discontinuation of treatment were tumor progression in 2 patients (14.3%) and peripheral neuropathy in 3 patients (21.4%) from the younger group versus 4 patients (50.0%) and 2 patients (25.0%), respectively, in the elderly group (P = 0.0963 and 0.6199 by Fisher's exact probability test). In the younger group, there was also 1 case of discontinuation after re-resection and 2 patients discontinued treatment due to hematological toxicity (a second dose reduction was necessary according to the criteria in Table 1).

### Response

Nineteen patients (12 from the younger group and 7 from the elderly group) could be evaluated for their response to treatment (Table 5). There were no patients with a complete response. The response rate was 60.0% in the younger group and 50.0% in the elderly group, while the disease control rate (PR+SD) was 100% and 83.3% in the younger and elderly groups, respectively. Thus, there was no difference of the response in relation to age.

**Table 5:** Antitumor Effects

	**< 70 Years (n = 14)**	≥ **70 Years (n = 8)**	P values*
**RR (%)**	60.0	50.0	0.5490
**DCR (%)**	100	83.3	0.3750
**CR/PR/SD/PD/NE**	0/6/4/0/2	0/3/2/1/1	-

Abbreviation: CR, complete response; PR, partial response; SD, stable disease; PD, progressive disease; NE, not evaluable; RR, response rate (CR+PR); DCR, disease control rate (CR+PR+SD).

*P values were calculated with the use of Fisher's exact probability test.

## Discussion

In 1957, 5-fluorouracil (5-FU) became available clinically, and the advent of 5-FU therapy [5,6] was followed by 5-FU/leucovorin (LV) therapy [7] that has remained standard chemotherapy for colon cancer for a very long time. After irinotecan and oxaliplatin became available, clinical studies including randomized comparative trials [8-10] of concomitant treatment with these agents and 5-FU/LV were performed. As a result, combination therapy with oxaliplatin + 5-FU/LV (FOLFOX4 or FOLFOX6) or with irinotecan + 5-FU/LV (FOLFIRI) has become standard chemotherapy for unresectable advanced/recurrent colorectal cancer in Western countries. Since the release of oxaliplatin in Japan in April 2005, FOLFOX therapy has rapidly become widespread, and it is described in the Guidelines for Management of Colon Cancer [3] (published in July 2005) as the standard therapy for unresectable advanced/recurrent colorectal cancer. FOLFOX4 therapy has thus become a standard therapeutic option for advanced/recurrent colorectal cancer in many countries. In addition, FOLFOX6 [11] therapy without bolus administration of 5-FU/LV on the second day has been developed to reduce adverse reactions and simplify treatment, and it is widely used as part of the trend for chemotherapy to be given on an ambulatory basis. Although the safety and efficacy of L-OHP+5-FU/l-LV therapy (original FOLFOX6) have already been investigated in Japan, little has been reported about mFOLFOX6 therapy, in which the dose of oxaliplatin is reduced to 85 mg/m^2 ^(the dose covered by the Japanese national health insurance scheme) [12]. In addition, there is still no standard therapy for elderly patients with colon cancer. Generally, the pharmacokinetics of drugs in elderly patients differs from those in younger patients due to decreased organ function associated with aging [13,14]. As a result, adequate treatment may not be provided to elderly patients compared with non-elderly patients due to fear of adverse drug reactions, and the examination of appropriate administration methods for the elderly has not been pursued adequately. In recent years, it has been confirmed that molecular-targeting drugs, including bevacizumab, are effective for colon cancer [15], and these drugs are already included as part of standard therapy in Western countries. Kabbinavar *et al*. reported that age had no influence on the safety of the combined administration of bevacizumab with 5FU-based chemotherapy [16], and concomitant use of a molecular-targeting drug that may be less toxic is expected to be a possible treatment option for elderly patients. Since the release of bevacizumab in Japan in June 2007, molecular targeting therapy has rapidly become widespread, however, concomitant use of bevacizumab is still often difficult in elderly patients because of concern about serious adverse events such as thrombosis and gastrointestinal perforation [15,17,18]. It is known that completing the administration of 5-FU/LV, irinotecan, and oxaliplatin according to the recommended schedule increases the survival time [19]. Thus, FOLFIRI and FOLFOX are still needed for combined therapy and it is considered extremely important to establish the safety of these regimens in elderly patients.

Accordingly, we examined the safety and efficacy of mFOLFOX6 therapy in elderly patients over 70 years old when the dose of oxaliplatin was reduced to 85 mg/m^2 ^(the dose covered by the national health insurance scheme).

Colorectal cancer is currently the third highest cause of cancer death and the second most common cancer in Japan. Since the average lifespan is currently 78.6 years for males and 85.6 years for females, a rapid increase of elderly patients with colorectal cancer is predicted in this country. Accordingly, it is problematic if elderly patients cannot receive effective chemotherapy simply because of their age, so the establishment of safe and effective standard therapy for elderly Japanese patients is important.

In Western countries, however, it is considered possible to treat the elderly with standard therapy, provided that the performance status (PS) is good, the function of major organs is maintained, and there are no uncontrolled complications. Goldberg *et al*. [20] reported that Grade 3/4 neutropenia and thrombocytopenia showed higher rates in elderly patients, but there were no differences of the response rate and safety of FOLFOX therapy between elderly patients over 70 years old and younger patients as a result of meta-analysis.

In present study, the elderly group was defined as patients more than 70 years old to assess the safety and efficacy of mFOLFOX6 therapy. We found that the incidence of Grade 3–4 neutropenia tended to be higher in elderly patients than younger patients, but there was no statistical significance (62.5% vs. 28.6%, P = 0.1347). Also, the incidence and severity of other adverse events in this study were generally comparable to those reported in Western countries [20]. The regimen was tolerable and there were no deaths due to toxicity.

When setting the dose-reduction criteria and the method of administration after occurrence of adverse events, it was decided that the dose of oxaliplatin would not be reduced, and that bolus 5-FU would be deleted due to the possibility that dose-limiting hematological toxicity such as neutropenia (which showed a high incidence in this study) might be caused by rapid intravenous injection of 5-FU [21-23]. After bolus 5-FU was stopped in accordance with the dose-reduction criteria (Table 1) due to grade 4 neutropenia in 3 patients (one younger patient and 2 elderly patients) during this study, treatment could be continued safely until PD occurred. Peripheral neuropathy is a characteristic adverse reaction to oxaliplatin and is the dose-limiting toxicity of this drug. Occurrence of neuropathy is dependent on the total dose of oxaliplatin, and grade 3–4 neuropathy (NCI-CTC criteria) shows an incidence of about 15% when the total dose reaches 750 to 800 mg/m^2^[24]. The dose-dependent neuropathy caused by oxaliplatin is reversible after suspension/omission of the drug, and treatment using a stop-and-go strategy (with reinstitution of therapy after recovery from toxicity) achieves favorable survival [25] and is well tolerated by elderly patients over 75 years old [26]. In the present study, neuropathy showed a lower incidence than that mentioned above, but there was a similar correlation between the total dose of oxaliplatin and the severity of neuropathy in both the younger and elderly groups (Figure 2).

Although the time to treatment failure (TTF) was 6.2 months in the younger group versus only 4.9 months in the elderly group, the number of treatment cycles was 10.0 and 9.5, respectively, showing that administration of mFOLFOX6 was possible in elderly patients with a good PS. The response rate was 60.0% in the younger group and 50.0% in the elderly group, while the disease control rate was 100% and 83.3%, respectively, showing no significant difference in relation to age.

When this study was initiated in San-in, a rural region of Japan with a large elderly population, there was an urgent need to establish effective chemotherapy regimens for colorectal cancer, which has recently become much more common in Japan. Accordingly, the present study was intended to assess the feasibility of mFOLFOX6 in Japanese colorectal cancer patients, including elderly patients, with regard to the incidence and severity of adverse events. In an attempt to rapidly investigate the efficacy and safety of mFOLFOX6, the subjects were enrolled during a 1-year period. The limited duration of enrollment resulted in too small a sample size for the study to be adequately powered. Despite this, our findings suggested that mFOLFOX6 is similarly tolerable and effective for elderly patients as it is for non-elderly patients, because the therapy could be administered at its recommended dosage without causing more severe adverse events than in non-elderly patients by employing appropriate criteria for patient selection, treatment suspension, and dose reduction in consideration of factors such as the PS and comorbidities.

However, discontinuation was necessary in 12 patients (including 3 elderly patients) because of adverse reactions, and 5 patients (including 2 elderly patients) discontinued treatment due to peripheral neuropathy (the dose-limiting toxicity of oxaliplatin). Therefore, avoiding or reducing the occurrence of such adverse events is necessary for the establishment of safer standard therapy.

## Conclusion

It was confirmed by the present study that mFOLFOX6 therapy, a standard chemotherapy for unresectable advanced/recurrent colorectal cancer, could be performed safely in elderly Japanese patients. The tolerability and efficacy of mFOLFOX6 therapy can be expected to be similar in the elderly, provided that the PS is good, the major organs are functioning well, and there are no uncontrolled complications.

The present findings also suggested that withdrawal of bolus 5-FU to avoid severe neutropenia might allow the continuation of treatment. Because discontinuation due to peripheral neuropathy (the dose-limiting toxicity of this regimen) was common, methods to avoid or alleviate such adverse events without reducing efficacy need to be investigated.

## Competing interests

The authors declare that they have no competing interests.

## Authors' contributions

AK, KK, HY, and MI conceived and designed the study, SS, AK, KK, HY, and HT collected and assembled the data, SS performed the statistical analysis, and SS wrote the manuscript. All authors have read and approved the final manuscript.
